# Massive Open Online Courses (MOOC) Evaluation Methods: Protocol for a Systematic Review

**DOI:** 10.2196/12087

**Published:** 2019-03-07

**Authors:** Kimberley Foley, Abrar Alturkistani, Alison Carter, Terese Stenfors, Elizabeth Blum, Josip Car, Azeem Majeed, David Brindley, Edward Meinert

**Affiliations:** 1 Global eHealth Unit Department of Primary Care and Public Health Imperial College London London United Kingdom; 2 Healthcare Translation Research Group Department of Paediatrics University of Oxford Oxford United Kingdom; 3 Department of Learning, Informatics, Management and Ethics Karolinska Institutet Stockholm Sweden; 4 Department of Primary Care and Public Health School of Public Health Imperial College London London United Kingdom

**Keywords:** online learning, e-learning, education, learning, education distance, computer-assisted instruction

## Abstract

**Background:**

Massive open online courses (MOOCs) have increased in popularity in recent years. They target a wide variety of learners and use novel teaching approaches, yet often exhibit low completion rates (10%). It is important to evaluate MOOCs to determine their impact and effectiveness, but little is known at this point about the methodologies that should be used for evaluation.

**Objective:**

The purpose of this paper is to provide a protocol for a systematic review on MOOC evaluation methods.

**Methods:**

We will use the Preferred Reporting Items for Systematic Reviews and Meta-Analyses Protocols (PRISMA-P) guidelines for reporting this protocol. We developed a population, intervention, comparator, and outcome (PICO) framework to guide the search strategy, based on the overarching question, “What methods have been used to evaluate MOOCs?” The review will follow six stages: 1) literature search, 2) article selection, 3) data extraction, 4) quality appraisal, 5) data analysis, and 6) data synthesis.

**Results:**

The systematic review is ongoing. We completed the data searches and data abstraction in October and November 2018. We are now analyzing the data and expect to complete the systematic review by March 2019.

**Conclusions:**

This systematic review will provide a useful summary of the methods used for evaluation of MOOCs and the strengths and limitations of each approach. It will also identify gaps in the literature and areas for future work.

**International Registered Report Identifier (IRRID):**

DERR1-10.2196/12087

## Introduction

Massive open online courses (MOOCs) are rapidly becoming an established method of online and distance education, growing in prominence since the launch of the first MOOC in 2008. The idea of a course accessible by anyone with a computer with no fees or prerequisites for joining has caught the attention and imagination of many involved in e-learning, with MOOC being called the educational buzzword of 2012 [1]. Numerous MOOCs have been developed by top universities such as Harvard, Stanford, and the Massachusetts Institute of Technology, giving additional gravitas to the field. MOOCs are accessible through multiple online platforms such as edX, Coursera, and FutureLearn. The possibility for anyone with a computer to participate in courses given by these universities and many other academic institutions has led to MOOCs being heralded as the democratization of education [2]. While traditional lectures are given to, at most, several hundred students, MOOCs have no participant limit and can potentially be given to tens of thousands of learners [3]. The scope of MOOCs is expanding beyond universities and into the workplace, with the flexible and self-directed nature of these courses making them highly transferable into the working environment. There is an increasing range of reasons for partaking in MOOCs, from mandatory university courses, to professional development, to self-interest [3].

While the MOOC field is new territory, the means of evaluating MOOCs is newer still and a gap in knowledge exists with regard to the methodologies which should be used for evaluation. The novel combination of teaching approaches used, including prerecorded videos, live discussion forums, peer-assessed assignments, and social media debate, warrant thorough investigation to enable providers to maximize participation and impact [4]. It is vital that appropriate methods are identified and available to determine the impact of these courses, a crucial but underresearched element. Aspects such as the effectiveness and quality of learning and impact of knowledge gained are vitally important in determining the strength of MOOCs as a learning tool, but there is not a substantial evidence base on methods for how these factors are measured or evaluated [5]. The longer-term impact of undertaking a MOOC must also be understood; at present there is little follow-up data gathered after the courses have concluded. This information is particularly needed when courses are designed to increase the knowledge or skills of a specific working population. Issues such as the almost universal low completion rates of MOOCs (ie, 10% or lower) are also in urgent need of addressing and improvements must be made to increase retention [6].

Although there have been recent reviews conducted on MOOCs [7-10], none have specifically focused on methods used for evaluation. With the heterogeneity of participants in MOOCs and the low retention rate [11], conducting effective evaluations of MOOCs is critical. To date, little work has been done in this area [12] and it has been highlighted as an area for future research [13]. Despite increasing research about MOOCs, there are limitations in reporting the methods and/or using valid and reliable measures in the studies [9]. Although it may not be advisable to develop a standard way to evaluate MOOCs due to their heterogeneity, a review on evaluation methods could help inform future evaluations on the current state of knowledge and the most reliable methods that can be used.

The purpose of this paper is to provide a protocol for a systematic review on MOOC evaluation methods. The systematic review is designed to identify all the relevant literature published thus far on methods of MOOC evaluation, extract methodologies and objectives, and synthesize these into a narrative describing the spectrum of methods available and recommendations for future research and practice.

## Methods

### Overview

We will follow elements of the Cochrane Handbook for Systematic Reviews for conducting the review [14] and will use Preferred Reporting Items for Systematic Reviews and Meta-Analyses Protocols (PRISMA-P) for reporting this protocol [15] (see Multimedia Appendix 1). To identify appropriate Medical Subject Headings (MeSH) and keywords, we will use the population, intervention, comparator, and outcome (PICO) framework to build the research question. We will follow six stages in this systematic review: (1) literature search, (2) article selection, (3) data extraction, (4) quality appraisal, (5) data analysis, and (6) data synthesis.

### Literature Searches

#### Inclusion and Exclusion Criteria

Course evaluations can have many definitions. In this review, we will focus on the definition by Edwards, which states that evaluations focus on the experience of teachers and the students to assess and illustrate their *effectiveness* [16]. Therefore, we will only include studies that focus on the evaluation of MOOCs with reference to the course design, materials, or topics. The studies will be included only if they were evaluating the MOOC in general or features directly related to MOOCs, such as MOOC videos, MOOC discussion posts, and MOOC assessments. We developed the following PICO framework to guide the search strategy, based on the overarching question, “What methods have been used to evaluate MOOCs?”:

1. Population: the target population will include learners in any geographic area who have participated in MOOCs.
2. Intervention: the intervention will be *MOOC evaluation methods*. This is intended to be broad to include qualitative, quantitative, and mixed methods.
3. Comparator: studies do not need to include a comparator for inclusion in this systematic review.
4. Outcome: learner-focused outcomes such as attitudes, cognitive changes, learner satisfaction, etc, will be assessed.

This PICO was converted to a search strategy with the assistance of a medical librarian, as shown in Table 1.

#### Inclusion Criteria

We will include studies with a primary focus on MOOC evaluation and studies that have applied or reviewed MOOC evaluation methods: quantitative, qualitative, or mixed. Evaluation of MOOCs does not need to be the primary focus of the paper for inclusion in this systematic review.

Publication dates will be restricted from 2008 to 2018. The start date of 2008 was selected because MOOCs were introduced in 2008 [17]. Studies from any geographic location will be included.

**Table 1:** PICO^a^ framework search strategy for MOOC^b^ evaluation methods.

Search categories	Sample search terms to screen within electronic databases
Phenomenon of interest	MOOC* OR “massive open online course” OR coursera OR edX OR odl OR Udacity OR futurelearn
Intervention	Evaluat* OR measur* OR compar* OR analys* OR report* OR assess*
Comparator	Method*
Outcome	Knowledge OR “applicable knowledge” OR retent* OR impact OR quality OR improv* OR environment OR effect

^a^PICO: population, intervention, comparator, and outcome.

^b^MOOC: massive open online course.

#### Exclusion Criteria

We will restrict publications to the English language only. Studies will also be excluded if the primary focus is e-learning or blended learning, but not MOOCs.

We will search the following databases: (1) Scopus, (2) Education Resources Information Center (ERIC); (3) Institute of Electrical and Electronics Engineers (IEEE) Xplore, (4) Medline/PubMed, (5) Web of Science, and (6) British Education Index. To identify potentially relevant grey literature, we will also search Google Scholar and Google search engines. The search strategy for Scopus was developed in consultation with a medical librarian. The search strategy was adjusted for the rest of the databases based on the keywords of each database. The complete search strategy is included in Multimedia Appendix 2. Search results will be imported into EndNote and duplicates removed.

### Screening and Article Selection

All records identified from the software searches will be recorded in a software management program, EndNote X8.2 (Clarivate Analytics). EndNote will also be used to remove any duplicates. Two independent reviewers will screen the title and abstract of all identified studies against the eligibility criteria. The full text of the identified studies will then be reviewed and assessed for eligibility. Disagreements will be resolved by discussion or by consultation with a third reviewer, if required.

Once the final list of studies is determined, the references for each included article will be searched to identify additional studies that should be considered for inclusion.

A PRISMA flow diagram will be created to document the selection process and reasons for article exclusions to ensure repeatability of the search results. This will include (1) Identification: records identified through database searching, additional records identified through other sources, and records after duplicates removed; (2) Screening (by title and abstract): including the number of records screened and records excluded; (3) Eligibility: full-text articles assessed for eligibility and full-text articles excluded, with reasons; and (4) Included: studies included in qualitative synthesis.

### Data Extraction

The full text of each manuscript will be reviewed and data extracted with data points as defined in Table 2. The first reviewer will complete the data abstraction table for each of the included studies; this form will then be reviewed by the second reviewer. We have kept the data extraction table fields in the free form because we have anticipated that there will be high heterogeneity between studies, which can limit the use of predetermined fields. However, we were able to create predetermined fields for the data collection method and evaluation method fields of the table (see Table 2) based on initial reading of MOOC evaluations. Subvariables related to the comparator may be added to the data extraction sheet based on the available information, such as comparator type and comparison data analysis method.

### Quality Appraisal

We will assess the quality of the included studies by conducting a risk of bias assessment. If there are any randomized controlled trials included, we will use the Cochrane Collaboration risk of bias tool [18]. Otherwise, for observational cohort and cross-sectional studies, we will use the National Institutes of Health-National Heart, Lung, and Blood Institute quality assessment tool [19]. The quality of the included studies will be recorded in a table for publication.

### Data Analysis

We do not expect to be able to conduct a meta-analysis due to the anticipated heterogeneity of studies. We will therefore summarize the data by conducting a descriptive analysis. To commence the analysis, we will compare the studies based on the evaluation method—quantitative, qualitative, or mixed methods—and data collection methods. We will include information on the evaluation methods, size of the groups of learners, characteristics of the learners, and description of the evaluation outcomes.

### Data Synthesis

We will also provide a narrative synthesis of the included studies. We will summarize the findings and present a table of the main results from all included papers. These will be supported by a narrative addressing the process as well as any rationale and challenges at each stage. These results will summarize and describe the MOOC evaluation methods, but also identify gaps and highlight areas where further research would be useful.

**Table 2:** Data extraction table.

Article information	Data extracted
General information	Article title Author name(s) Publication date Country of origin
Study characteristics	Study aims and rationale Study research question(s) Theoretical underpinning
Methods	Research design Outcome measure(s) Data collection method (yes/no) Survey: precourse survey, postcourse survey, other survey Interview Learning management system data Discussion posts Quizzes: pretest, posttest, other test, quiz Data analysis model/method Main data analysis method Secondary data analysis method
Intervention (evaluation method)	Type of learner(s) Evaluation method: quantitative, qualitative, mixed methods Group size
Outcome measures	Learner-focused outcomes (eg, knowledge, skills, and attitude/behavior) Other outcomes (eg, cost-effectiveness and other)
Comparator details	(If applicable)

## Results

The systematic review is ongoing. We completed the data searches and data abstraction in October and November 2018. We are now analyzing the data and expect to complete the systematic review by March 2019. We will submit the findings for publication and peer review.

## Discussion

This systematic review will provide a *systematic and transparent* review of the literature in order to better understand the strengths and weaknesses of methods currently used to evaluate various aspects of MOOCs. The key implications drawn from the synthesized data will help to inform future evaluation work. In this section, any researcher assumptions will be discussed, as well as conclusiveness of the data; strengths, weaknesses, and limitations of the systematic review; gaps in the current literature; and possibilities for future research.

## Appendix

Multimedia Appendix 1 PRISMA-P 2015 Checklist.

Multimedia Appendix 2 Full search strategy.

Multimedia Appendix 3 Peer-reviewer report from EIT Health.

## Abbreviations

EIT: European Institute of Innovation and Technology
ERIC: Education Resources Information Center
IEEE: Institute of Electrical and Electronics Engineers
MeSH: Medical Subject Headings
MOOC: massive open online course
PICO: population, intervention, comparator, and outcome
PRISMA-P: Preferred Reporting Items for Systematic Reviews and Meta-Analyses Protocols
