# Supplementary material for: Massive Open Online Courses (MOOC) Evaluation Methods: Protocol for a Systematic Review
Source: JMIR Res Protoc. 2019 Mar 7;8(3):e12087. doi: 10.2196/12087 (PMC6427096; doi:10.2196/12087)
Supplement: Multimedia Appendix 1 [file resprot_v8i3e12087_app1.pdf]

Multimedia Appendix 1

Table displaying the PRISMA-P 2015 Checklist: table adapted from Table 2 in Shamseer, L. *et al.* (2015) 'Preferred reporting items for systematic review and meta-analysis protocols (PRISMA-P) 2015: elaboration and explanation', *BMJ*, 349, p. g7647. doi: 10.1136/bmj.g7647.

Section and Topic	#	Checklist Item	Information reported		Page number(s)
			Yes	No	
**Administrative information**					
Title: identification	1a	Identify the report as a protocol of a systematic review	☒	☐	1
Title: Update	1b	If the protocol is for an update of a previous systematic review, identify as such	☐	☒	
Registration	2	If registered, provide the name of the registry (such as PROSPERO) and registration number	☐	☒	
Authors: Contact	3a	Provide name, institutional affiliation, e-mail address of all protocol authors; provide physical mailing address of corresponding author	☒	☐	1
Authors: Contributions	3b	Describe contributions of protocol authors and identify the guarantor of the review	☒	☐	8
Amendments	4	If the protocol represents an amendment of a previously completed or published protocol, identify as such and list changes; otherwise, state plan for documenting important protocol amendments	☐	☒	
Support: Sources	5a	Indicate sources of financial or other support for the review	☒	☐	8
Support: Sponsor	5b	Provide name for the review funder and/or sponsor	☒	☐	8
Support: Role of Sponsor or Funder	5c	Describe roles of funder(s), sponsor(s), and/or institution(s), if any, in developing the protocol	☒	☐	8

Section and Topic	#	Checklist Item	Information reported		Page number(s)
			Yes	No	
**Introduction**					
Rationale	6	Describe the rationale for the review in the context of what is already known	☒	☐	3
Objectives	7	Provide an explicit statement of the question(s) the review will address with reference to participants, interventions, comparators, and outcomes (PICO)	☒	☐	4
**Methods**					
Eligibility criteria	8	Specify the study characteristics (such as PICO, study design, setting, time frame) and report characteristics (such as years considered, language, publication status) to be used as criteria for eligibility for the review	☒	☐	4-5
Information sources	9	Describe all intended information sources (such as electronic databases, contact with study authors, trial registers or other grey literature sources) with planned dates of coverage	☒	☐	5
Search strategy	10	Present draft of search strategy to be used for at least one electronic database, including planned limits, such that it could be repeated	☒	☐	12-13
Study records: data management	11a	Describe the mechanism(s) that will be used to manage records and data throughout the review	☒	☐	5
Study records: selection process	11b	State the process that will be used for selecting studies (such as two independent reviewers) through each phase of the review (that is, screening, eligibility and inclusion in meta-analysis)	☒	☐	5-6
Study records: data collection process	11c	Describe planned method of extracting data from reports (such as piloting forms, done independently, in duplicate), any processes for obtaining and confirming data from investigators	☒	☐	5-6

Section and Topic	#	Checklist Item	Information reported		Page number(s)
			Yes	No	
Data items	12	List and define all variables for which data will be sought (such as PICO items, funding sources), any pre-planned data assumptions and simplifications	☒	☐	4
Outcomes and prioritization	13	List and define all outcomes for which data will be sought, including prioritization of main and additional outcomes, with rationale	☒	☐	4,6
Risk of bias in individual studies	14	Describe anticipated methods for assessing risk of bias of individual studies, including whether this will be done at the outcome or study level, or both; state how this information will be used in data synthesis	☒	☐	7
Data synthesis	15a	Describe criteria under which study data will be quantitatively synthesised	☐	☒	
	15b	If data are appropriate for quantitative synthesis, describe planned summary measures, methods of handling data and methods of combining data from studies, including any planned exploration of consistency (such as I2 , Kendall's τ)	☐	☒	
	15c	Describe any proposed additional analyses (such as sensitivity or subgroup analyses, meta-regression)	☐	☒	
	15d	If quantitative synthesis is not appropriate, describe the type of summary planned	☐	☒	
Meta bias(es)	16	Specify any planned assessment of meta-bias(es) (such as publication bias across studies, selective reporting within studies)	☐	☒	
Confidence in cumulative evidence	17	Describe how the strength of the body of evidence will be assessed (such as GRADE)	☐	☒	

Multimedia Appendix 2

Search strategy

Scopus:

(TITLE-ABS-KEY (mooc* OR "massive open online course" OR coursera OR edx OR odl OR udacity OR futurelearn) AND TITLE-ABS-KEY (evaluat* OR measur* OR compar* OR analys* OR report* OR assess*) AND TITLE-ABS-KEY (knowledge OR "applicable knowledge" OR retent* OR impact OR quality OR improv* OR environment OR effect "learning outcome" OR learning))

Limit 2008 to present
Results: 1489

Ovid:

(MOOC Or massive open online course OR coursera or Udacity or futurelearn OR edx and MOOC And EVALUATION STUDIES/ OR evaluat$ or measur* or compar* or analys* or report* or assess*) And (knowledge OR KNOWLEDGE/ OR Educational measurement/ or learning outcome/ or recent or impact or quality or improv" or environment or effect OR learn or Learning/)

Limit 2008 to present
Results: 65
Notes: The Ovid search did not include edx alone because it resulted in a large number of irrelevant studies related to cells.

ERIC:
(mooc* OR "massive open online course" OR coursera OR edx OR odl OR udacity OR futurelearn)

Limit 2008 to present
Results: 1131
Notes: The ERIC search was kept more general than other databases because adding more terms significantly limited the search.

Web of science:

Indexes=SCI-EXPANDED, SSCI, A&HCI, CPCI-S, CPCI-SSH, ESCI Timespan=All years
# 1 TS=(evaluate* OR measure* OR compare* OR analys* OR report* OR assess*)

2 TS=(Knowledge OR “applicable knowledge” OR retention OR impact OR quality OR improve OR environment OR effect OR participation OR completion OR learning OR learn)
3 TI=(MOOC or "Massive open online course")
4 88 Ti=(coursera OR odl OR udacity OR futurelearn)
5 1,199 #4 OR #3
6 #5 AND #2 AND #1

Limit 2008 to present
Results: 479

British Education Index

(mooc* OR "massive open online course" OR coursera OR edx OR odl OR udacity OR futurelearn) AND (evaluate* OR measure* OR compare* OR analys* OR report* OR assess*) AND (Knowledge OR “applicable knowledge” OR retention OR impact OR quality OR improve OR environment OR effect OR participation OR completion OR learning OR learn)

Limit 2008 to present
Results: 111
