# Supplementary material for: Massive Open Online Courses (MOOC) Evaluation Methods: Protocol for a Systematic Review
Source: JMIR Res Protoc. 2019 Mar 7;8(3):e12087. doi: 10.2196/12087 (PMC6427096; doi:10.2196/12087)
Supplement: Multimedia Appendix 2 [file resprot_v8i3e12087_app2.pdf]

Multimedia Appendix 2

Search strategy

Scopus:

(TITLE-ABS-KEY (mooc* OR "massive open online course" OR coursera OR edx OR odl OR udacity OR futurelearn) AND TITLE-ABS-KEY (evaluat* OR measur* OR compar* OR analys* OR report* OR assess*) AND TITLE-ABS-KEY (knowledge OR "applicable knowledge" OR retent* OR impact OR quality OR improv* OR environment OR effect "learning outcome" OR learning))

Limit 2008 to present
Results: 1489

Ovid:

(MOOC Or massive open online course OR coursera or Udacity or futurelearn OR edx and MOOC And EVALUATION STUDIES/ OR evaluat$ or measur* or compar* or analys* or report* or assess*) And (knowledge OR KNOWLEDGE/ OR Educational measurement/ or learning outcome/ or recent or impact or quality or improv” or environment or effect OR learn or Learning/)

Limit 2008 to present
Results: 65
Notes: The Ovid search did not include edx alone because it resulted in a large number of irrelevant studies related to cells.

ERIC:
(mooc* OR "massive open online course" OR coursera OR edx OR odl OR udacity OR futurelearn)

Limit 2008 to present
Results: 1131
Notes: The ERIC search was kept more general than other databases because adding more terms significantly limited the search.

Web of science:

Indexes=SCI-EXPANDED, SSCI, A&HCI, CPCI-S, CPCI-SSH, ESCI Timespan=All years
# 1 TS=(evaluate* OR measure* OR compare* OR analys* OR report* OR assess*)
# 2 TS=(Knowledge OR “applicable knowledge” OR retention OR impact OR quality OR improve OR environment OR effect OR participation OR completion OR learning OR learn)
# 3 TI=(MOOC or "Massive open online course")

4 88 Ti=(coursera OR odl OR udacity OR futurelearn)
5 1,199 #4 OR #3
6 #5 AND #2 AND #1

Limit 2008 to present
Results: 479

British Education Index

(mooc* OR "massive open online course" OR coursera OR edx OR odl OR udacity OR futurelearn) AND (evaluate* OR measure* OR compare* OR analys* OR report* OR assess*) AND (Knowledge OR “applicable knowledge” OR retention OR impact OR quality OR improve OR environment OR effect OR participation OR completion OR learning OR learn)

Limit 2008 to present
Results: 111
