# Supplementary material for: Massive Open Online Courses (MOOC) Evaluation Methods: Protocol for a Systematic Review
Source: JMIR Res Protoc. 2019 Mar 7;8(3):e12087. doi: 10.2196/12087 (PMC6427096; doi:10.2196/12087)
Supplement: Multimedia Appendix 3 [file resprot_v8i3e12087_app3.pdf]

EIT Health e.V.
Mies-van-der-Rohe-Str. 1 C
80807 Munich | Germany
German tax registration number 143/213/31212

T: +49 (0)151 1636 9604
E: ursula.muehle @eithealth.eu
W: eithealth.eu

Munich, 30 October 2018

To:
Edward Meinert
The Chancellor, Masters and Scholars of the University of Oxford 0040
edward.meinert@paediatrics.ox.ac.uk

Dear Edward,

Congratulations! Your proposal "Real World Evidence" with the proposal ID "19562" has been approved for continuation in the 2019 EIT Health CAMPUS Portfolio!

All Innovative Education proposals went through strict eligibility checks and intensive face-to-face evaluations with external experts. These steps were important in selecting outstanding proposals for our 2019 Business Plan. Your proposal has passed all stages of the selection process, something that you and your cross-EU team can feel proud of.

As part of the final step in this process and before your project may officially join the 2019 BP we would like you to make some changes to your proposal. These changes are based on the feedback from Fall Review evaluation board. We have compiled a step-wise approach below. Please follow them accurately, in order, to ensure that your proposal is of top quality and can thus be approved by EIT to join the 2019 Business Plan.

As a reminder, the Project Management Office is here to support you:

**Campus projects**	**Oana Neagu**	**oana.neagu@eithealth.eu**

These changes need to be done by: **14th November 2018**

EIT Health is supported by the EIT,
a body of the European Union

Please inform all your co-applicants about EIT Health's decision and any requirements for changes to be made. If you have any questions, please do not hesitate to contact us. We thank you for your efforts and look forward to working with you in 2019!

Yours sincerely,

Ursula Mühle
Director of Education

TECHNICAL PROCEDURE TO AMEND 2019 PROPOSALS:

DEADLINE 14th November 2018

STEP 1: EIT Health Plaza

The proposal has been reopened in Plaza.

Access is under the tile BP 2019. All tabs are open for you to amend based on the evaluator feedback at the end of this letter.

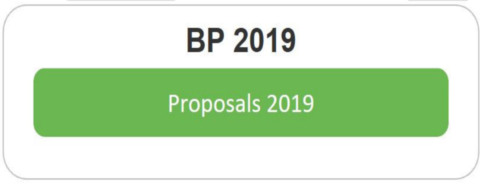

STEP 2: Changes in Plaza

Below, you will find the changes to be made. Please make these changes to the corresponding tabs in Plaza.

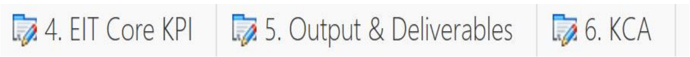

As a reminder, there is a guidebook for filling the 2019 proposals available on Connections as well as webinars and slides. These are found under Guidance for Partners at https://connections.eithealth.eu/documents/21826/0/2019+EIT+Health+Call+for+Proposals+-+Plaza+Guidebook/a4c89f4f-5ba7-94c8-0263-3b12af3d0e9d.

STEP 3: Submit

Once you are done, please submit your proposal.

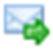

Deadline: 14th November 2018.

Scoring and Feedback Report/Mandatory Changes/Recommendations

All eligible CAMPUS proposals were evaluated following the evaluation process. You will find the aggregated feedback in this section. You will also find some changes /revisions for your budget.

Please read this through carefully and in its entirety to adequately address the changes to your proposal.

Evaluator Feedback

Project Excellence and Strategic Fit

What is appreciated as a strong point of the project is its innovative approach to capture real-time data to improve disease management.

It should be kept in mind that the registry development aspect is a multi-year project.

Implementation and Impact in 2018

For the first MOOC, the target group should be looked into again.

It would be interesting to have a global perspective in HSV granular data.

It may be useful to consider looking at how other healthcare sectors use data to address diagnostics and treatment.

The number of KPIs should be increased.

Implementation and Feasibility in 2019

There may be an issue with the course coherence as the data has to be first collected before the trainees learn how to test it.

The question remains whether the face-to-face learning experiences for different cohorts are going to be consistent, as the case studies change over time.

The project looks very promising to be implemented also in the future.

It would be useful to explore telemedicine also as a communication and delivery tool.

The biobanks in the UK could be used as a case study.

The target audience for the course should be more carefully chosen/specified.

Impact & Sustainability in 2019

An advantage of the project is that the team is already engaged with an industry partner. Other partners from EIT could still be engaged.

The data sets should be integrated. In order to build on the data availble for the project, Genomics England could be involved.

The project may be very useful in helping prescribing medicines in a patient genetics sensitive fashion.

Mandatory Changes

Please reconsider your KPIs in Plaza, Call for Proposals 2019, Tab 3. Please assesses whether KPI EITN04   # Start-ups created will be attained by December 2019. Please bear in mind that to prove the attainment of this KPI documentation is required (e.g registration certificate, document such as an invoice or an online sales record certifying a first financial transaction for a service/product sold to a customer).

Please reconsider your outputs and whether they are in fact deliverables and vice-versa. Deliverables are a tangible document, medium, or other artefact encapsulating the quantifiable outputs. Outputs are rather intangible outcomes of a project like a service, method, design, concept, platform, event. Please update your milestones. These are significant points in the development of the activity (e.g. 30st August finalization recruitment of students). Milestones should not have as expected date December 2019. This can be done in Plaza, Call for Proposals 2019, Tab 5.

Budget Changes

N/A

Finance Recommendations

N/A

Director Recommendations

It would be useful to explore telemedicine also as a communication and delivery tool.

The biobanks in the UK could be used as a case study. In order to build on the data availble for the project, Genomics England could be involved.

The question remains whether the face-to-face learning experiences for different cohorts are going to be consistent, as the case studies change over time.

The target audience for the course should be more carefully chosen/specified.

Scoring

The total maximum score for an individual proposal is 100 points.

The final score of your proposal:

78.00

The maximum grant awarded for the entire activity for 2019 will be:

€249,589.00

EIT Health is supported by the EIT,
a body of the European Union

Guidance on budgets and suggested revisions

On top of the mandatory changes, there are some specific budget items that need your attention and that should be addressed. This is to make sure that we will have few questions by the EIT and compliant budgets:

1. External Project Partners (or Sub-grantees) and Sub-contractors

External Project Partners (or Sub-grantees)

Bringing in a non-KIC partner is allowed per EIT approval if there is clear need for this expertise within the project and not found within the consortium. Many of you have requested budget for such third parties. For proper implementation of these third parties in the Business Plan, please do the following:

1. External Project Partners should be registered in Plaza so that these can be listed in the Business Plan for EIT to approve. If this has not been done, please contact your project manager (see page 1 of this letter) to properly register these entities with EIT.

2. External Project Partners need to be specifically added as a separate budget line in Plaza's tab 7. To do this, simply create a new budget line with the registered external project partner (see 1. If not yet registered) and add to the cost category 4174 - C1 Sub-granting the amount that the KIC partner intends to allocate to the third party. The maximum amount allocated to external project partners via sub-granting is limited to 50,000€ per third party per year. Please make sure to not exceed this limit.

3. Furthermore, sub-granting should involve a pre-selection! You should document the selection of the external partners properly and duly. Further instructions on the handling of sub-granting will follow in due time.

Subcontracting

Please note that any subcontracting must of course follow your internal procurement and as such must be based on best value for money. The best value for money principle does NOT in all cases require competitive selection procedures. However, if you will not request several offers, you must document the selection procedure and how best value for money was ensured.

Therefore, please clarify in tab 8 "Budget Justification" the justification in your choice / pre-selection steps. If the third party has not yet been identified, please also indicate this here.

2. KCA

Please check the description of the KCA in tab 6. We have seen some projects with a too short description of the KCA (e.g. "*H2020 project*"). Please provide us with a short but meaningful description of the activity and the link to the KAVA (if not obvious).

Best practice (example):

ICanHear is an EU International Training Network programme focused on training fellows and developing knowledge to improve performance with hearing instruments such as cochlear implants (CI). The KCA focuses on recording neural responses in and developing stimulation patterns for users with two CIs (binaural). It is a required element to support the development of the training programs of the KAVA project.

3. Budget Justification

Please review your Budget Justification in tab 8. This is to give the EIT transparency. For your convenience, we have prepared a short "guidance note" (see annex at the end of this document) containing a brief description of the cost categories, details on what we are asking for and some best practice examples.

For **sub-granting**, please see above.

If you have included **co-funding**, you are kindly requested to provide a brief explanation for this as well. We would like to ask you to consider the amount of co-funding you have entered. It is essential that this co-funding is really made available. Please provide us with a short description of the nature of the co-funding (description field has been added). Please briefly explain the source of the co-funding.

4. Co-funding

Co-funding is defined as the difference between the total KAVA costs and the EIT grant. Some activities are not entirely financed by EIT contribution (reimbursement rate < 100%). In this case co-funding is an important part of the success of your activity. Please ensure that the co-funding is correctly calculated and added to the total cost of the activity. Furthermore, please note that co-funding cannot be reduced during the BP year, unless the overall EIT contribution is reduced.

5. NOTE regarding Associate Partners in your Activity

Associate Partners in EIT Health have a 250k€ threshold of the EIT Contribution, meaning these partners cannot receive more than 250k per business plan year. Some of these partners have received a separate notification regarding this limit, and as a consequence, these partners may come back to you with certain budget shifts. Thanks in advance for taking care of this.

IMPORTANT:

The below parts of your proposal are directly imported to the Business Plan (Annex I) and we know that EIT scrutinizes this information. Thus, it is a good idea to have these parts as concise and accurate as possible. You can still revise them at this stage in Plaza.

- **Activity Description**: Is this a crisp and concise summary of your activity/project?

- **Elevator Pitch**: Does this grasp in 2-3 short sentences the gist of your activity/project?
- **Work plan / work packages**: If your project spans over several years, the specific scope of work to be implemented **for the year 2019** shall be clear by explaining: (1) which specific tasks / work packages are planned for year 2019 and (2) which is/are the critical milestone(s) and DELs / OUTs to be achieved in year 2019. Are the work plans realistic and directly supporting the project's implementation, management, and follow-up steps? Are the work packages developed to meet key Milestones, supply quality Deliverables, and achieve KPIs?
- **KPIs**: Are they realistic? Do they complement your activity/project? Are they measurable?
- **Budgeting**: Is the budget reflective of the true costs of your activity/project? Does it properly reflect the involvement of all KIC partners and third parties?

Annex: Description of cost categories / Examples

(general note: KAVA costs need to comply with cost eligibility criteria of Horizon 2020)

Cost category	*Additional clarification on the type of cost*	*Required information*	*Best practice / Example for the description to be entered in Plaza – Please be as brief as possible*
**4000 - Personnel**	Personnel costs are eligible only if they are in accordance with the normal policy and practices of the partner (costs include the gross cost for the employer, i.e. the net salary to the employee + potential obligatory tax / social security payments). For direct personnel cost, the estimated FTE per each profile (e.g. manager, officer, assistant) shall be indicated forming the basis of the personnel cost calculation.	Number of FTE /<br><br>Job profile /<br><br>Average monthly costs	*1 FTE / manager / av. monthly costs of EUR 7.500*<br><br>*0.5 FTE / scientist /av. monthly costs of EUR 3.500*
**4172 - Sub-contracting**	A subcontractor is normally a third party which is not a KIC partner or a subsidiary of a KIC partner. Subcontracting occurs when a KIC partner procures services to carry out part of the KAVA, usually for	Expected value of the subcontract /<br><br>Name of subcontractor, if	*EUR 20.000 / LAB AG / laboratory testing of samples / existing framework contract*

	specialised tasks that a KIC partner cannot carry out itself or because it is more efficient to use the services of a specialised organisation.<br><br>Subcontracting may cover only a limited part of the action.	known (e.g. framework contract) /<br><br>Brief summary of the work performed by the subcontractor /<br><br>Awarding procedure	*EUR 60.000 / unknown / provision of testing samples / to be awarded by public bidding process*
**4174 - Sub-granting**	The KIC partners may give financial support to third parties. The recipient of such a support is a third party, i.e. a beneficiary which is not a KIC partner/linked entity. In the case of EIT Health these are external project partners or participants in programmes that receive a scholarship, travel grant, etc. As opposed to subcontracting, the recipient of the financial support does not necessarily deliver goods or provide services to the KIC partner. No profit element is involved, the financial support shall cover only the costs associated with the participation in the KAVA.	Amount of sub-granting /<br><br>Brief description of the selection of the grantee /<br><br>Name of grantee (if known) /<br><br>Description of activity that is funded and associated tasks	*EUR 25.000 / grantee: Amios AG / for participation in the project tasks (WP X, description of contribution to the tasks)*
**4175 - Prizes**	KIC partners may give prizes to third parties. Prizes may not be awarded directly without a contest, and shall be published by means of communication which are non-discriminatory in nature for the submission of entries and which have no effect on restricting the access of participants to the contest.<br><br>Please note: If you are awarding a scholarship for example, you	Nature of prize(s) /<br><br>Nature of contest	*Inducement prize announced at the beginning of the action for identifying a (new) approach to dealing with a technical implementation problem to be tackled at the end of the action.*

	should rather choose the "Sub-Granting" Category.		
**4100 - Travel and subsistence**	Actual costs of travel, accommodation and subsistence allowance relating to the implementation of KAVA are eligible	Number of trips /<br><br>Short description of major meetings, workshops, events /<br><br>Average costs per trip (suggestion maximum amount per trip: EUR 1.000) / no. of persons travelling	*Expected number of trips - 10 trips / 5 consortium meetings, 3 conferences, 2 workshops / expected EUR 750 per trip / 1 person*
**4135 - Depreciation**	The depreciation costs of equipment, infrastructure or other assets as recorded in the beneficiary's accounts are eligible.<br><br>Only the portion of depreciation costs of the fixed assets corresponding to the duration of the KAVA and the rate of actual use for the purposes of the KAVA is eligible provided that it is written off in accordance with the tax and accounting rules applicable	Amount of depreciation /<br><br>Description of asset /<br><br>Asset depreciation range /<br><br>Expected use within the project	*EUR 2.000 / IT server / standard depreciation 5 years / use in project: 1 years*<br><br>*EUR 1.500 / sample heater machine / standard deprecation 7 years / use in project 1 year*
**4141 - Cost of other goods and service**	Direct costs having a short life expectancy, such as consumables, office supplies and sundry items, etc.	Description of the good or service /<br><br>Number and costs per good or service	*Specialized software to evaluate research results / EUR 2.000*<br><br>*Translation services / EUR 7.500*
**4210 - Co-funding**	Co-funding in this case means the partners' co-payment to cover the full KAVA costs of the project	Short description of nature and source of co-funding	*Co-payment of partner in order to cover full KAVA costs*
